# The combination of tibial anterior translation and axial rotation into a single biomechanical factor improves the prediction of patient satisfaction over each factor alone in patients with ACL reconstructed knees

**DOI:** 10.1007/s00167-017-4497-7

**Published:** 2017-03-15

**Authors:** Thomas P. Branch, Shaun K. Stinton, William C. Hutton, Philippe Neyret

**Affiliations:** 1 University Orthopedics, Decatur, GA USA; 2 ArthroMetrix LLC, 441 Armour Place NE, Atlanta, GA 30324 USA; 3 Department of Orthopaedics, Emory University School of Medicine, Atlanta, GA USA; 4 Department Orthopedic Surgery, Centre Albert-Trillat, Hôpital de la Croix-Rousse, Lyon, France

**Keywords:** ACL, Knee laxity, Joint play envelope, ACL reconstruction, Rotational knee laxity, Anterior knee laxity, Patient satisfaction

## Abstract

**Purpose:**

The purpose of this study was to identify biomechanical factors, in both reconstructed and healthy knees, that correlate with patient satisfaction after ACL reconstruction.

**Methods:**

Seventeen patients who had undergone unilateral ACL reconstruction were reviewed 9 years post-op. Patients completed subjective questionnaires and underwent manual knee laxity testing (Lachman-Trillat, KT-1000, and pivot shift) and automated laxity testing. During automated testing, both legs were rotated into external rotation and then internal rotation until peak rotational torque reached 5.65 Nm. Load-deformation curves were generated from torque and rotation data. Features of the curves were extracted for analysis. Total leg rotation and anterior laxity during KT-1000 testing were combined into a single factor (Joint Play Envelope or JPE). Patients were divided into groups based on patient satisfaction scores (Group 1: Higher Satisfaction, Group 2: Lower Satisfaction, Group 3: Unsatisfied). Load-deformation curve features and manual laxity testing results were compared between groups 1 and 2 to determine which biomechanical factors could distinguish between the groups. Diagnostic screening values were calculated for KT-1000 testing, the pivot shift test, total leg rotation and JPE.

**Results:**

During manual testing, no significant differences in biomechanical factors were found when comparing reconstructed knees in group 1 and group 2. When comparing the reconstructed and healthy knees within group 2, the reconstructed knees had a significantly higher displacement during the KT-1000 manual maximum test (*p* < 0.002). When considering the reconstructed knees alone, neither the result of the pivot shift test nor KT-1000 testing could distinguish between group 1 and group 2. During automated testing, there were no significant differences between the groups when comparing the reconstructed lower limbs. The healthy lower limbs in group 2 had more maximum external rotation (*p* < 0.02) and decreased stiffness at maximum external rotation (*p* < 0.02) when compared to the healthy lower limbs in group 1. Total leg rotation was unable to distinguish between group 1 and group 2. JPE could distinguish between group 1 and group 2 when considering the reconstructed limb alone (*p* < 0.02). All four diagnostic screening values for JPE were equal or higher than in the other criteria. JPE also showed the most significant correlation with patient satisfaction.

**Conclusions:**

Joint Play Envelope is an objective measure that demonstrated improved predictive value as compared to other tests when used as a measure of satisfaction in patients with ACL reconstructed knees.

## Introduction

Outcome analysis, with an emphasis on patient satisfaction, has been of increased interest in sports medicine since Kocher et al. published a study in 2004 that described the relationship between ligament stability and subjective assessment of knee function [11]. In that study, patient satisfaction after anterior cruciate ligament (ACL) reconstruction was shown to be significantly correlated with the grade of the pivot shift test during the manual clinical knee examination and not the amount of anterior tibial translation during the Lachman-Trillat test. In the decades prior to the Kocher study, surgeons had focused primarily on correcting increased anterior tibial translation by reducing the positive Lachman-Trillat findings present in an ACL deficient knee. Multiple devices were developed to quantify anterior translation in order to demonstrate objective changes in the knee after an ACL tear and after reconstruction [8, 20].

Many surgeons during the 1960s and 1970s believed that the ACL deficient knee was best characterized by rotational instability [17, 22]. After the 2004 paper by Kocher et al., there was a sudden shift of community interest towards the pivot shift test to incorporate rotational instability into the knee examination since the test represents a coupling of rotatory and translational instability [14]. While the pivot shift test remains the sine qua non of the symptomatic ACL deficient knee, extensive work on its standardization has been required to improve its performance as a consistent outcome predictor [7, 9, 10, 15, 16, 18]. Joint Play Area (JPA) is a single biomechanical factor that combines both absolute translational and rotational instability within the knee and may be able to provide similar information as the pivot shift test. The concept of JPA was originally defined as a combination of total tibiofemoral rotation and anterior–posterior translation [5, 21]. However, it may be more descriptive to rename the term “joint play area” and call it “joint play envelope” (JPE) since it is a three-dimensional measure rather than a two-dimensional measure.

The aim of this study was to identify biomechanical factors that correlate with patient satisfaction after single-bundle ACL reconstruction. The primary hypothesis was that biomechanical factors exist in both ACL reconstructed knees and healthy knees that correlate with patient satisfaction and that these factors could be used to predict outcome scores. It was further hypothesized that rotational and translational characteristics could be combined into a single factor (Joint Play Envelope, or JPE) that would provide a better prediction of patient satisfaction scores than one single characteristic. It is important to clarify that success or failure of these ACL reconstructed knees was not the aim of this study.

## Materials and methods

Seventeen patients who had undergone unilateral bone-patellar tendon-bone ACL reconstruction were retrospectively reviewed an average of 9 years after surgery (range: 8–10 years). All necessary approvals with regard to the testing of patients were obtained at the institution where the testing was carried out. All surgeries were performed by a single author between January 1998 and May 1999. The ACL reconstruction technique used the middle third of the patellar tendon with one bone block press fit in the femur and another bone block fixed in the tibia with an interference screw. Extra-articular augmentation, when performed, consisted of a gracilis tendon autograft routed through a hole in the femoral bone block, with both limbs passed under the lateral collateral ligament and attached via bone tunnels on either side of Gerdy’s tubercule with the knee in neutral rotation and flexed to 30°.

At the time of review, each patient completed three validated subjective questionnaires: the Knee Injury and Osteoarthritis Outcome Score (KOOS), the International Knee Documentation Committee subjective score (IKDC), and a modified Visual Analog Scale (VAS). The modified VAS utilized a horizontal seven inch line with a vertical line indicating 0 at one endpoint and 100 at the other endpoint. The patient was asked to place a vertical line between the two endpoints indicating their satisfaction with the outcome between 0 and 100% satisfied. The location between the two endpoints was used to determine the patient’s satisfaction score. Physical examination was performed by two independent orthopaedists (not the treating physician). Each physician performed manual knee laxity tests (Lachman-Trillat and Pivot Shift) and instrumented knee laxity tests (KT-1000 performed at 67 N, 89 N, 133 N and manual maximum force) [8]. The manual maximum force was the force applied during the manual maximum tibial displacement test. All tests were performed in a blinded and randomized fashion.

**Fig. 1:**
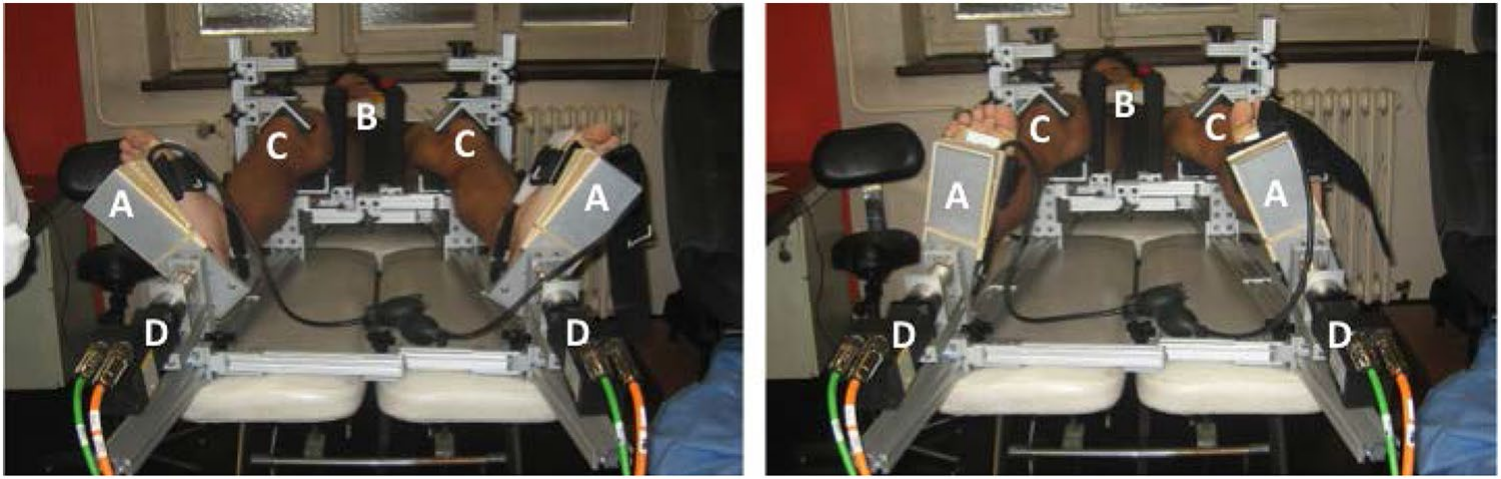
The robotic lower leg axial rotation system showing a patient lying supine on the testing table whose feet are strapped into footplates (*a*), with both femurs stabilized using distal femoral posts (*b*), and both patellae locked into the trochlear grove with clamps (*c*) as torque is applied through the use of servomotors (*d*) during external rotation testing (*Left*) and internal rotation testing (*Right*)

Each subject then underwent standardized, automated lower limb laxity testing using the system shown in Fig. 1 [2–6, 21, 23]. The term “lower limb laxity testing” is used because the version of the device used in this study applies force to the foot using a motor and records rotational motion using the same motor. This rotation of the footplate rotates the foot and then the lower leg, so it would not be accurate to report the laxity as knee laxity rather than lower limb laxity. The behaviour of the entire system of the lower limb is important in understanding knee stability since it determines the interaction between the knee and the ground. Subsequent versions of the automated device allow for measurement of tibial motion, thereby making quantification of knee laxity possible in addition to lower limb laxity. In the current study, both lower limbs were placed into the device and tested simultaneously using previously described methods [6]. Both legs were rotated into external rotation until the peak rotational torque equaled 5.65 Nm, at which point the direction of rotation reversed into internal rotation and rotation continued until the same torque was reached. Overall internal and external rotation of the operative and healthy leg was compared utilizing previously described methods. The measurement accuracy of the system was within 0.01° and 0.001 Nm of torque as defined by the manufacturer of motors (Baldor Electric Company, Fort Smith, AR).

**Fig. 2:**
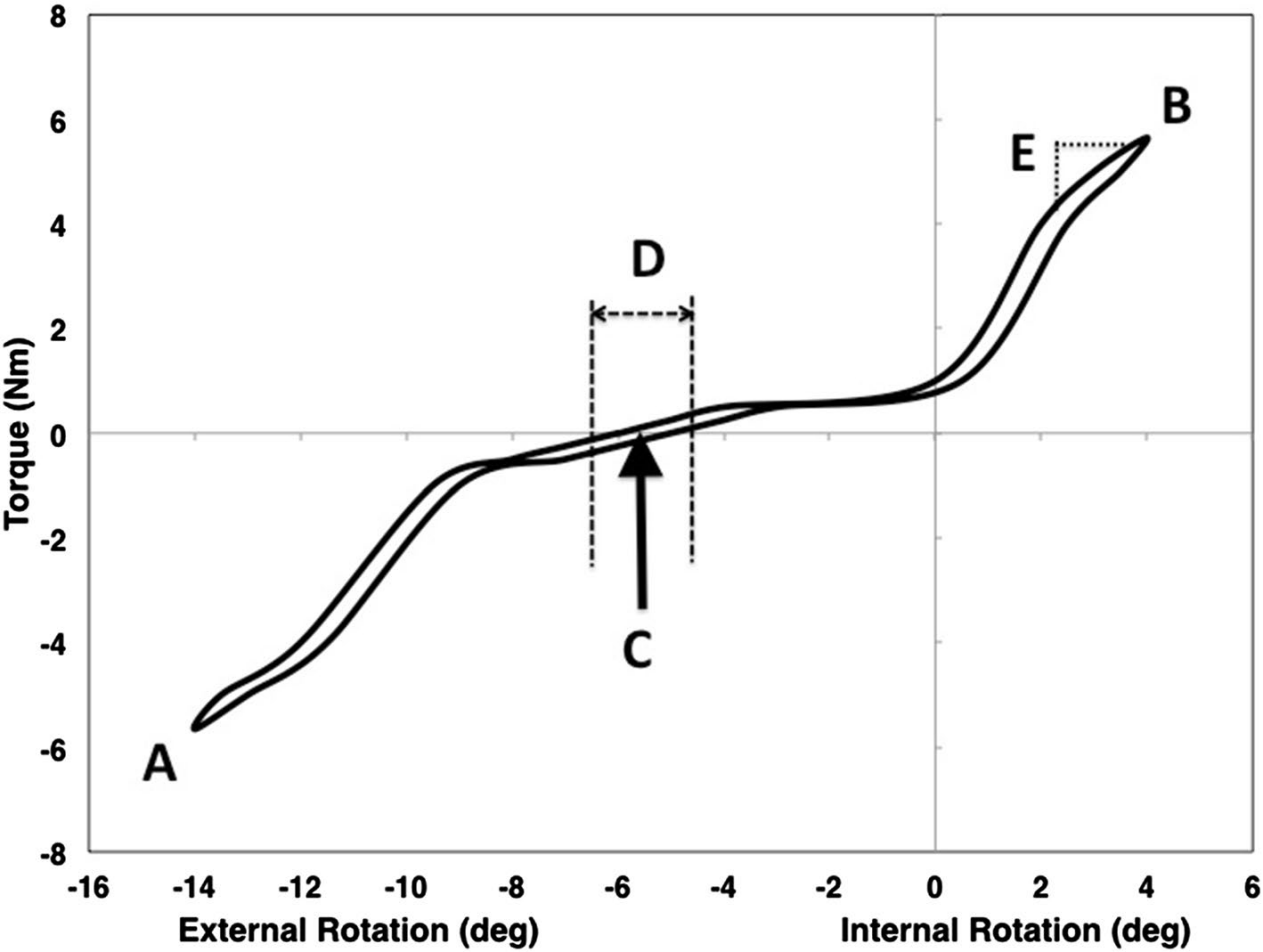
An example torque-angular deformation curve showing the extracted features including the maximum external rotation (*a*), maximum internal rotation (*b*), rotational position at 0 Nm of torque (*c*), the amount of play at 0 Nm of torque (*d*) and the endpoint slope (*e*)

Load-deformation curves were generated from the torque and rotation data as described previously [6]. Features of the load-deformation curves including maximum external rotation, maximum internal rotation, the rotational position at 0 Nm of torque, the amount of play at 0 Nm of torque and endpoint slope over the last 10% of the curve were extracted for comparative analysis (Fig. 2). A higher percentage change, or steeper slope, represents a less compliant limb (stiffer limb), whereas a lower percentage change, or less steep slope, represents a more compliant limb (less stiff limb) in response to rotational torque. The amount of play at 0 Nm of torque was determined by the width of the hysteresis curve at that point (Fig. 2).

In addition, total leg rotation (i.e. maximum external rotation plus maximum internal rotation, in degrees) and anterior laxity during KT-1000 testing using manual maximum force (mm) were combined into a single factor. This single factor was defined as the total leg rotation multiplied by the KT manual maximum which resulted in units of mm-deg. This has been defined in the past as “Joint Play Area” or JPA. In this study, we refer to this factor as the more appropriate term “Joint Play Envelope” (JPE) to represent its three-dimensional nature.

For comparison, the seventeen patients were divided into three groups based on VAS patient satisfaction scores. In previous research by Kocher et al., patient satisfaction was chosen as an ordinal number between 1 and 10 [11]. We chose VAS so as to form a continuous variable rather than ordinal for better statistical comparison. Group 1 (Higher Satisfaction: 9 patients) had satisfaction scores equal to or greater than 80. Group 2 (Lower Satisfaction: 6 patients) had satisfaction scores less than 80 and greater than 50. Group 3 (Outlier Patients: 2 patients) had satisfaction scores that were under 50 and represented two cases of post-operative arthrofibrosis. Statistical analysis was performed between Group 1 and Group 2 using standard tests in the R statistical software package (R: A language and environment for statistical computing. R Foundation for Statistical Computing, Vienna, Austria) with analysis of the load-deformation curves using pointwise *t* tests. Patient data for Group 1 and Group 2 are presented in Table 1. Since there are only two patients in Group 3, these patients are described individually. They are included here for observational interest and not for analysis.

**Table 1:** Patient information including demographic data, operative procedures and the presence of osteoarthritis for Group 1 (Higher Satisfaction) and Group 2 (Lower Satisfaction)

	Group 1	Group 2	*p* value
Sex			
Male	8	2	n.s.
Female	1	4	n.s.
Height (m)	1.75	1.68	n.s.
Weight (kg)	74.4	65.3	n.s.
Lateral tenodesis	2	3	n.s.
Meniscectomy			
Lateral	1	1	n.s.
Medial	4	3	n.s.
Osteoarthritis			
Patellofemoral	0	1	n.s.
Lateral	0	0	n.s.
Medial	1	2	n.s.

The results were categorized by group (Group 1: Higher Satisfaction vs. Group 2: Lower Satisfaction) and within each group into reconstructed and healthy knees/limbs. All patients in both groups were satisfied with their healthy, uninjured knee and reported their healthy knee to represent 100% satisfaction on the VAS. Paired data comparisons were applied when the analysis was between the reconstructed and healthy knees/limbs of one subject. Unpaired data comparisons were applied when limbs from Group 1 were compared to knees/limbs from Group 2.

IRB approval was not required at the institute where the study was performed. However, informed consent was obtained for all subjects.

### Statistical analysis

In order to provide the clinician with the best statistics available to ‘diagnose’ the unsatisfied patient in this study, all diagnostic screening values as reported by Altman and Bland were calculated [1]. These descriptors of a diagnostic test are sensitivity, specificity, positive predictive value and negative predictive value. The Fisher Exact Test was the primary statistic used to evaluate each diagnostic test. The intent was to provide the clinician with biomechanical values that allowed for classification of patients as opposed to simple correlations which only show statistical relationships. A power analysis was performed, and it showed that a sample size of 12 subjects would allow for a difference between groups of 1 mm or 1° to be identified with >80% power and 95% confidence.

## Results

### Manual knee examination results

**Fig. 3:**
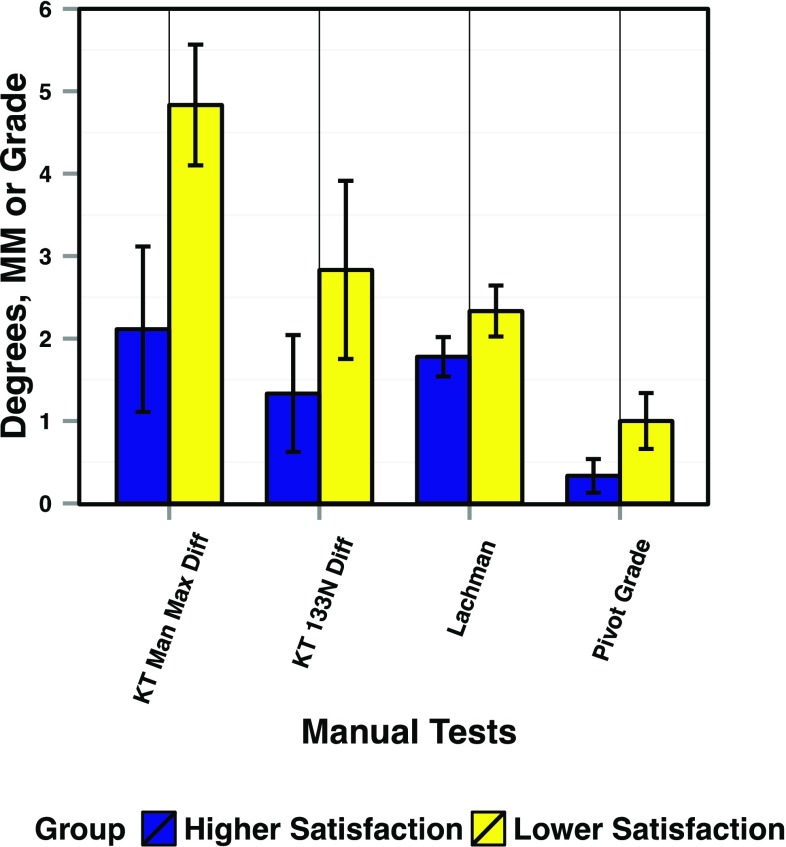
Comparison of measures from the manual clinical knee exam between Group 1 (Higher Satisfaction) and Group 2 (Lower Satisfaction). KT Man Max Diff is the side-to-side difference during KT-1000 testing at manual maximum force. KT 133 N Diff is the side-to-side difference during KT-1000 testing at 133 N

**Fig. 4:**
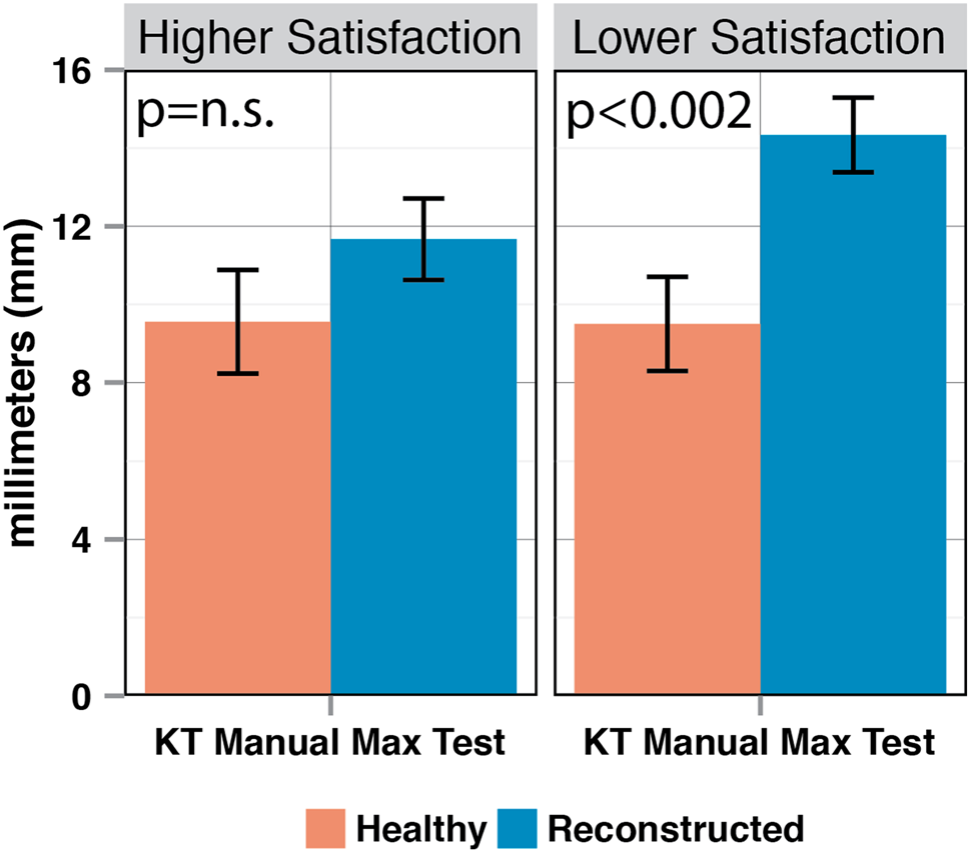
Side-to-side differences between the healthy knees and reconstructed knees during KT-1000 testing at manual maximum force for the Higher Satisfaction Group and the Lower Satisfaction Group

When comparing the manual knee examination results of the reconstructed knees in the Higher Satisfaction group (Group 1) with the reconstructed knees in the Lower Satisfaction group (Group 2), all measurements/grades were higher in Group 2 than in Group 1; however, no single comparison reached statistical significance (Fig. 3). When comparing the reconstructed knees in both groups with their healthy knee counterpart, only the Lower Satisfaction group (Group 2) had a significantly higher displacement during the KT-1000 manual maximum test than the healthy knees (*p* < 0.002) (Fig. 4). No significant differences within each group were seen in any other manual test.

**Fig. 5:**
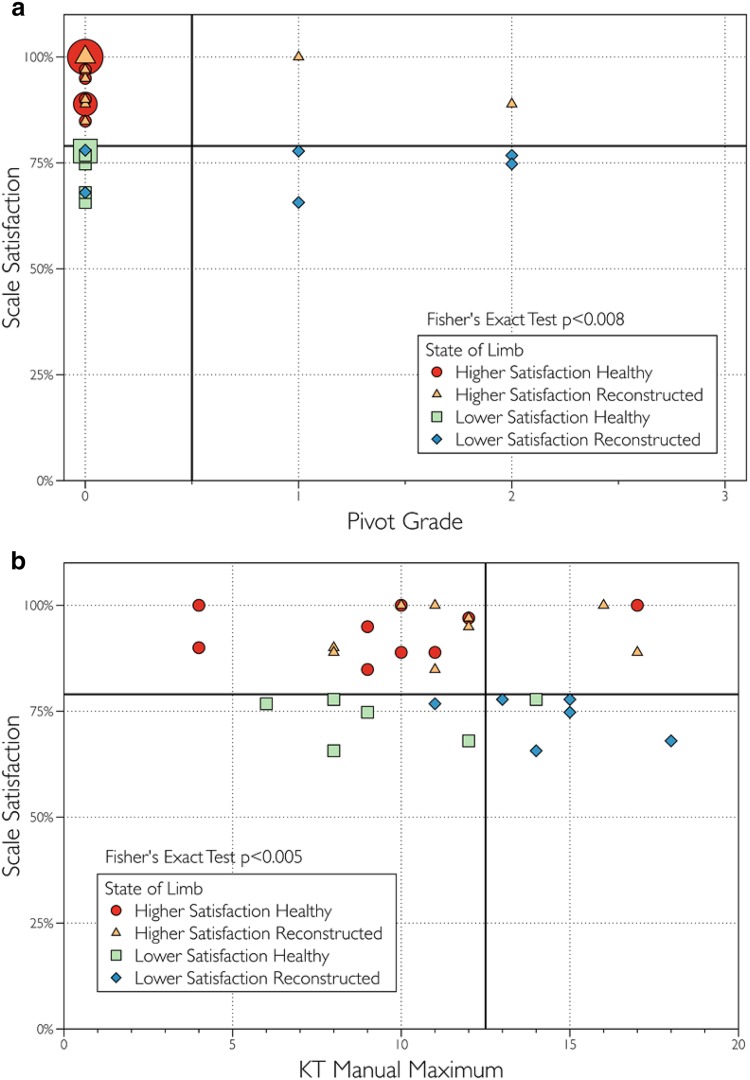
**a** The presence of a pivot shift alone can predict lower satisfcation with a limb when all knees (healthy and reconstructed) are included in the analysis (*p* < 0.01); **b** The KT-1000 test at manual maximum force can predict lower satisfaction with a limb when all knees (healthy and reconstructed) are included in the analysis (*p* < 0.01). The smallest symbols indicate a single subject, the medium sized symbols represent 2 subjects and the largest symbols represent 3 subjects

When all knees from both groups (healthy and reconstructed) were included in the analysis, both the presence of a positive pivot shift and results of KT-1000 testing at manual maximum force were able to distinguish between knees from the Higher Satisfaction group and the Lower Satisfaction group (*p* < 0.01) (Fig. 5a, b). However, when only the reconstructed knees were included in the analysis, neither the presence of a positive pivot shift nor results of KT-1000 testing at manual maximum force were able to distinguish between the two groups at a statistically significant level.

### Robotic testing results

When comparing the features of the load-deformation curves between the reconstructed and the healthy lower limbs within each group, there were no significant differences in maximum internal rotation, the rotational position at 0 Nm of torque, the amount of play at 0 Nm of torque, Total Leg Rotation or slope. There was a significant difference at maximum external rotation in the Lower Satisfaction group where the reconstructed lower limb had 8.7° less external rotation than the healthy lower limb (*p* < 0.04). There were no significant differences between the reconstructed lower limbs when comparing the two groups. However, there were significant differences between the two groups when comparing the healthy lower limbs. The healthy lower limbs in the Lower Satisfaction group had more maximum external rotation (64.5° vs. 48.7°, *p* < 0.02) and decreased stiffness at maximum external rotation (1.3 vs. 1.6, *p* < 0.02) when compared to the healthy lower limbs in the Higher Satisfaction group.

**Fig. 6:**
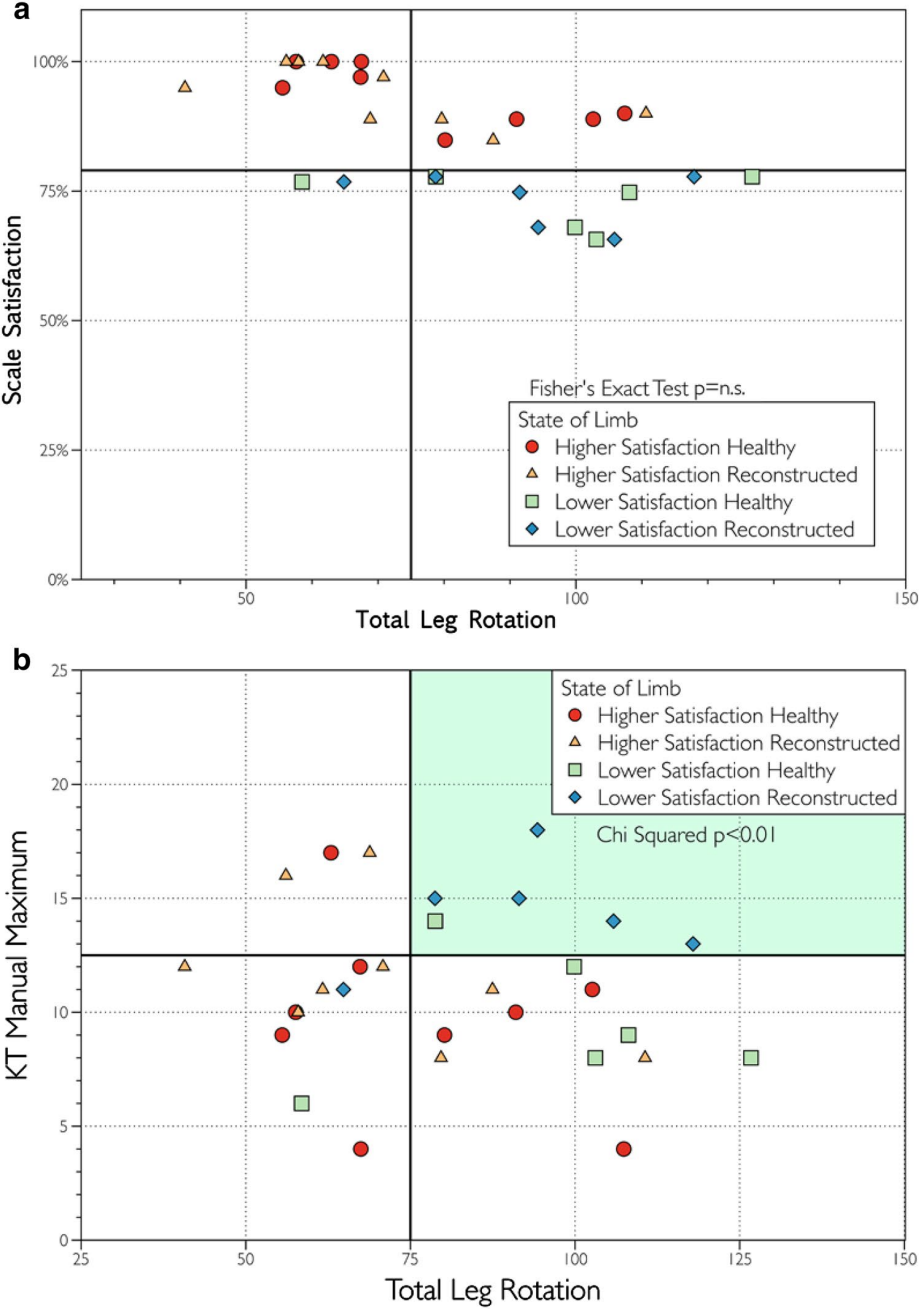
**a** Total Leg Rotation alone could not predict lower satisfaction with a limb (n.s.);**b** By plotting KT Manual Maximum vs Total Leg Rotation, clear statistically significant clustering can be seen allowing for prediction of lower satisfaction with a limb (*p* < 0.01)

When all lower limbs from both groups (reconstructed and healthy) were included in the analysis, total leg rotation was unable to distinguish between the lower limbs in the Higher Satisfaction group and the Lower Satisfaction group (n.s) (Fig. 6a). When comparing the reconstructed lower limbs between the two groups, there were no significant differences between the groups; however, there was a trend towards a higher total leg rotation in the reconstructed lower limbs in the Lower Satisfaction group when compared to the total leg rotation in the reconstructed lower limbs in the Higher Satisfaction group (92.2° vs. 70.4°, *p* < 0.057). This same comparison using the healthy limbs in both groups was not significant (95.8° vs. 76.9°, n.s.). On average, the reconstructed knees demonstrated 2.9° more total leg rotation in the Lower Satisfaction group than the Higher Satisfaction group.

**Fig. 7:**
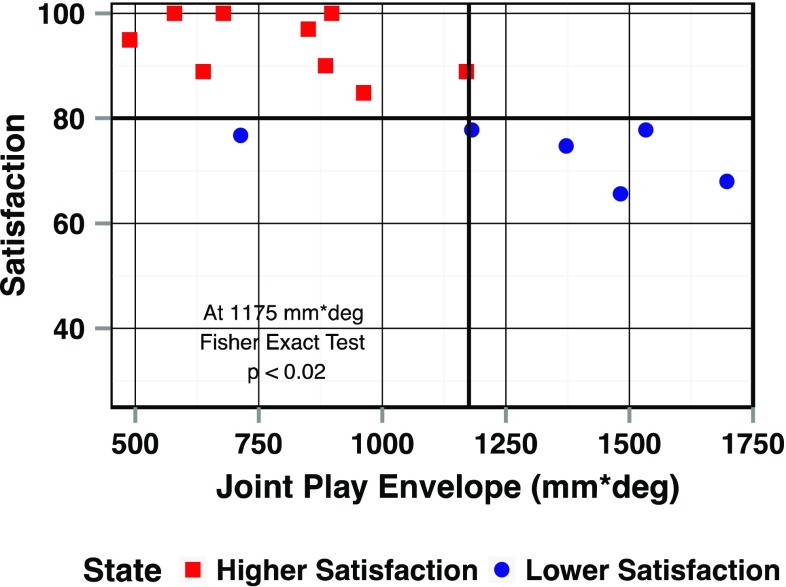
Joint Play Envelope can distinguish between the Higher Satisfaction group and the Lower Satisfaction group when looking at the reconstructed limbs alone (*p* < 0.02)

When total leg rotation and KT-1000 manual maximum results were combined, statistically significant clustering was identified (*p* < 0.01) (Fig. 6b). Joint play envelope as a single factor could distinguish between the two groups when focusing on the reconstructed lower limb alone (*p* < 0.02) (Fig. 7). Diagnostic screening values including sensitivity, specificity, positive predictive value, and negative predictive value are reported in Table 2 for criteria including KT-1000 testing using manual maximum force, pivot shift test result, total leg rotation and JPE. All four diagnostic screening values for JPE were equal or higher than in the other criteria. The higher sensitivity achieved using JPE vs the other criteria would result in reduced false-negative tests. Specificity was high (>90) among all four criteria. The pivot shift had the lowest positive predictive value, while the other three criteria had equivalent values. JPE had the highest negative value with pivot shift testing close behind. JPE also showed the most significant correlation with patient satisfaction.

**Table 2:** Diagnostic screening values are reported for: KT-1000 testing using manual maximum force; pivot shift test; total leg rotation and JPE

Criteria	Sensitivity	Specificity	Positive predictive value	Negative predictive value	*p* value
KT manual maximum test	56	95	83	83	0.005
Pivot shift test	67	92	67	92	0.008
Total leg rotation	29	92	83	59	n.s.
Joint play envelope	83	96	83	96	< 0.001

All four diagnostic screening values for JPE were equal or higher than in any of the other criteria

### Group 3: outlier patients

Group 3 consisted of two patients with very low VAS satisfaction scores of 20.2 (Patient 1) and 34.3 (Patient 2). Patient 1 had a reconstructed knee with six degrees less rotation but three millimetres more anterior translation than his limb with the healthy knee. He complained of a stiff knee with a sensation of increased movement when rising from a seated position or climbing stairs. The second unsatisfied patient (Patient 2) was the only patient to experience severe graft site pain after surgery. He had a negative pivot shift and the KT-1000 manual maximum side-to-side difference was 2 mm. He had a full, symmetrical range of motion from 0° to 150°.

## Discussion

The most important finding in this study is that the Joint Play Envelope is an objective measure that demonstrated excellent predictive value when used as a measure of satisfaction in patients with ACL reconstructed knees, as demonstrated by the diagnostic screening values reported in Table 2. JPE is calculated from a combination of rotation and translation, which may allow for improved repeatability and reliability of the measurements since the components are recorded from objective single-plane testing. Furthermore, the ability of a biomechanical measure to uniquely classify or identify the group of Lower Satisfied Patients in this study is a diagnostic improvement over correlation or association alone.

Musahl et al. published clinical guidelines for the pivot shift examination in order to improve the consistency of the results. Even though his guidelines for standardization of the pivot shift have significantly improved the grading process, variability in testing remains an issue [16]. There have been attempts to instrument the pivot shift with varying degrees of success. In a review article published in 2013, 28 in vivo studies and 41 in vitro studies were identified that measured at least one quantitative parameter in the analysis of the pivot shift test [13]. Twenty-five different parameters were used to quantify the pivot shift. The methods varied greatly between studies and the lack of agreement makes standardization of a methodology difficult. To date, the reproducibility, reliability and accuracy data of these instrumented techniques are unknown.

The grading of any manoeuvre during a physical examination is subjective. Surgeons grading the pivot shift in their own post-operative patients may be a source of bias. In this study, we used two independent surgeons to grade the pivot shift. While it is inconvenient to have two examiners evaluate patient knees for the presence of the pivot shift in any prospective study, it is important to have at least one independent surgeon perform the test. The fact that the pivot shift specificity remains high but the sensitivity was low suggests that, for this study, the criteria for suggesting a pivot shift was high. In other words, both independent surgeons may have agreed to a stricter description of a pivot shift which excluded the subtle “Pivot Glide” or grade I pivot shift. In order to have a positive pivot shift, the tibia needed to significantly sublux during the examination. This would have resulted in a lower sensitivity but a higher specificity for the test.

The pivot shift remains a fundamental test for the presence of a symptomatic knee after an ACL reconstruction. If an examiner can elicit a positive pivot shift, thereby mimicking a subluxation of the knee, it is an indication that the reconstruction in not performing in the desired manner. The appeal of the pivot shift test also relates to its side-to-side independence. The presence of a positive pivot shift correlates with reduced satisfaction in that knee. The issues with the pivot shift test revolve around the consistency and comfort of the examination itself along with the difficulty in objectively quantifying the results.

While the success or failure of these ACL reconstructions was not the focus of this study, it is interesting to note that total leg rotation as a component of JPE demonstrated a trend towards higher values in the Lower Satisfaction group as compared to the Higher Satisfaction group. This suggests that increased rotation in the injured limb may not have been managed by surgical intervention. In addition, the operative limb in Group 2 demonstrated a statistically significant reduction in maximum external rotation while maintaining the same total leg rotation and external rotation endpoint stiffness as the healthy limb. This suggests that the operative limb in Group 2 patients had surgical results creating the ‘pre-positioning’ of the tibia into external rotation described in a previous paper. This ‘pre- positioning’ results in a limitation of internal rotation of the tibia with respect to the femur which may be a cause of reduced patient satisfaction [6].

A statistically significant association between residual pivot shift and patient satisfaction after ACL reconstruction has been reported previously [11, 12]. While association between two features is an important statistical method in research, the ability to use data to classify patients into groups makes for an improved means of diagnosis. Since the pivot shift is a coupled rotational and translational manoeuvre, selection of one feature with the exclusion of the other may be problematic. In fact, it is important to note that statistically significant classification could not occur with KT or Total Leg Rotation alone. It is the combination of the two factors that allows for statistically significant classification as shown by the diagnostic screening values. However, it appears that a baseline high total leg rotation may be somewhat predictive of a less satisfied patient. This is even true when using the healthy limb as a predictor of satisfaction. Therefore, Joint Play Envelope is an objective measure that demonstrated improved predictive value as compared to other tests when used as a measure of satisfaction in patients with ACL reconstructed knees. Benefits of using JPE to characterize the knee include its clinician-independent capture, and like the pivot shift, its side-to-side independence. Potential sources of error in the acquisition of three-dimensional motion between the femur and tibia during robotic testing of the knee have been described [2]. When testing bias is minimized, results can be consistent across days of testing. The increased reliability, reproducibility, accuracy, and precision of testing should result in improved diagnostic screening values. In this study, the use of the JPE as a diagnostic screening test showed increased sensitivity for patient satisfaction over the other tests.

This study has several limitations. The number of patients was small. Despite this small sample size, excellent statistical predictability was achieved. This may be attributable to the use of the automated testing system. While there are a number of uneven confounding factors between the two groups such as sex, associated injuries and the use of an extra-articular tenodesis, the aim of the study was to use biomechanical factors to identify patients with lower satisfaction. This was accomplished despite the uneven distribution. A number of patients had extra-articular tenodesis in addition to ACL reconstruction. Pain associated with the tenodesis or additional graft harvest, as well as the additional lateral scar, could all influence patient satisfaction. There was an uneven gender distribution between the groups. The effect of sex on patient satisfaction is unknown. Whilst a recent systematic review found no gender difference in patient reported outcome measures following ACL reconstruction, satisfaction was not independently considered [19].

## Conclusions

Joint Play Envelope is an objective measure that demonstrated improved predictive value as compared to other tests when used as a measure of satisfaction in patients with ACL reconstructed knees. Surgical decision making could be affected in patients identified as being at risk for being unsatisfied after ACL reconstruction with additional lateral reinforcement or the use a double-bundle graft rather than a single-bundle graft in these patients.
